# Plasma Cystatin C as a Marker of Acute-on-Chronic Liver Failure and 28-Day Mortality in Patients With Acutely Decompensated Liver Cirrhosis: A Prospective Observational Study

**DOI:** 10.7759/cureus.116426

**Published:** 2026-09-17

**Authors:** Faizudheen P, Deepak K Saini, Pooja Gunwal, Shashikant Paswan, Manisha B Thakur

**Affiliations:** 1 Department of Medicine, Vardhman Mahavir Medical College & Safdarjung Hospital, New Delhi, IND; 2 Department of Orthopaedics, Maharishi Chyawan Government Medical College &amp; Rao Tularam Hospital, Koriawas, IND; 3 Department of Orthopaedics, Vardhman Mahavir Medical College & Safdarjung Hospital, New Delhi, IND; 4 Department of Anaesthesiology, Maharishi Chyawan Government Medical College &amp; Rao Tularam Hospital, Koriawas, IND; 5 Department of Anaesthesiology, Vardhman Mahavir Medical College & Safdarjung Hospital, New Delhi, IND; 6 Department of Physical Medicine and Rehabilitation, Vardhman Mahavir Medical College & Safdarjung Hospital, New Delhi, IND

**Keywords:** acute-on-chronic liver failure, biomarkers, cystatin c, glomerular filtration rate, liver cirrhosis

## Abstract

Background: Acute decompensation of cirrhosis carries a 28-day mortality of 1.9%, rising to nearly 30% with acute-on-chronic liver failure (ACLF), in which renal failure is the most frequent and most ominous organ failure. Serum creatinine overestimates glomerular filtration rate in cirrhosis. Plasma cystatin C may better reflect renal function and prognosis, but Indian data are scarce. We assessed cystatin C as a marker of ACLF and 28-day mortality in acutely decompensated cirrhosis.

Methods: In this prospective observational cohort study, 100 adults with acutely decompensated cirrhosis were enrolled over 18 months. We performed clinical assessments and standard investigations, and measured plasma cystatin C by nephelometric immunoassay within 48 hours of admission. Participants were followed for ACLF and 28-day mortality. Cystatin C, Child-Turcotte-Pugh (CTP), and Model for End-Stage Liver Disease (MELD) scores were compared as predictors of mortality by receiver operating characteristic (ROC) curve analysis.

Results: ACLF developed in 41 participants (41%), and 44 (44%) died within 28 days. Mean plasma cystatin C was higher in non-survivors than survivors (5.49±4.70 vs 2.06±1.54 mg/L; p<0.001) but did not differ by ACLF status (p=0.271). Cystatin C predicted 28-day mortality with AUROC 0.781 (95% CI 0.688-0.874); a cut-off ≥2.5 mg/L gave 73% sensitivity and 75% specificity, outperforming CTP (0.506) and MELD (0.508) scores (p<0.001).

Conclusions: Plasma cystatin C rose in patients who died within 28 days and predicted short-term mortality better than CTP or MELD scores, although it did not discriminate ACLF status. Cystatin C may serve as a simple, reproducible adjunct for prognostication in decompensated cirrhosis; larger multicentric studies are needed to validate this cut-off.

## Introduction

Cirrhosis typically evolves from a silent, compensated stage into a decompensated one, announced by ascites, variceal haemorrhage, hepatic encephalopathy or jaundice. Most patients who decompensate acutely still have a reasonably favourable short-term outlook, with roughly 1.9% dying within 28 days; that figure climbs to almost 30% once acute-on-chronic liver failure (ACLF) sets in [1]. Renal failure is the most common organ failure in ACLF and is associated with a worse prognosis than the failure of any other single organ [2].

Serum creatinine remains the most widely used marker of renal function because it is inexpensive and easily available, but its concentration is confounded by reduced muscle mass, low dietary protein intake and increased tubular secretion in cirrhosis, so that it systematically overestimates the glomerular filtration rate (GFR) in this population [3,4]. Cystatin C, a low-molecular-weight cysteine-protease inhibitor produced by all nucleated cells at a constant rate and cleared almost exclusively by glomerular filtration, is a more sensitive marker of change in GFR than creatinine and correlates better with directly measured (inulin) clearance in patients with liver cirrhosis [5,6]. It rises within 12 to 24 hours of renal injury, earlier than creatinine, and is less influenced by muscle mass, diet, and bilirubin levels.

In a large prospective European cohort, baseline plasma cystatin C independently predicted renal dysfunction, hepatorenal syndrome, ACLF and mortality in patients with acute decompensation of cirrhosis [7]. Data on plasma cystatin C in Indian patients with acutely decompensated cirrhosis are, however, scarce. The primary objective of this study was to assess plasma cystatin C as a predictor of 28-day mortality in patients with acute decompensation of cirrhosis, including its performance relative to the Child-Turcotte-Pugh (CTP) and Model for End-Stage Liver Disease (MELD) scores. The secondary objective was to determine whether cystatin C level was associated with the development of ACLF. Note: Cystatin C was not evaluated and is not proposed here as a diagnostic test for ACLF itself.

## Materials and methods

This prospective, hospital-based observational cohort study was conducted over 18 months in the Department of General Medicine of a tertiary-care teaching hospital in New Delhi, India, along with the Department of Pathology. The study was approved by the Institutional Ethics Committee of Vardhman Mahavir Medical College & Safdarjung Hospital (approval no. IEC/VMMC/SJH/Thesis/2019-10/55). Every participant gave written informed consent before enrolment.

We screened consecutive adults (aged over 18 years) admitted for acute decompensation of cirrhosis, identified by new-onset or worsening ascites, hepatic encephalopathy, or gastrointestinal bleeding in someone with known cirrhosis, and excluded anyone with pre-existing renal disease or malignancy. All patients meeting these criteria during the enrolment period were included consecutively. None were excluded for missing data. A total of 100 participants completed the 28-day follow-up. The sample size was calculated from a prior report of cystatin C's diagnostic performance (73.3% sensitivity and 52.5% specificity for short-term mortality; 58.0% sensitivity and 66.4% specificity for ACLF) [7] at 80% power, a two-sided α of 0.05 and 15% allowable precision. This required at least 84 participants, so we enrolled 100 to build in a margin.

Every participant had a detailed history and examination, abdominal ultrasonography, and venous blood drawn within 48 hours of admission for a complete blood count, renal and liver function tests, serum electrolytes, random blood glucose, viral markers (HBsAg, anti-HCV, HIV), and plasma cystatin C. Cystatin C was quantified by particle-enhanced immunonephelometry on a BN II System analyser (Siemens Healthineers, Forchheim, DEU) using a 2 mL ethylenediaminetetraacetic acid (EDTA) sample per the manufacturer's assay protocol. Fasting was not required as cystatin C production and clearance are not materially affected by recent food intake. The GFR was estimated from the 2012 Chronic Kidney Disease Epidemiology Collaboration (CKD-EPI) equation that combines creatinine and cystatin C [8].

We applied the Asian Pacific Association for the Study of the Liver (APASL) consensus definition of ACLF, i.e., an acute hepatic insult presenting with jaundice (serum bilirubin above 5 mg/dL) and coagulopathy (international normalised ratio (INR) above 1.5), progressing to ascites and/or hepatic encephalopathy within four weeks in someone with chronic liver disease that may or may not have been recognised previously [9]. Consistent with the chronic liver failure-sequential organ failure assessment (CLIF-SOFA) renal subscore, a serum creatinine above 1.5 mg/dL was used to indicate renal dysfunction [1,10]. Severity of cirrhosis was graded using the CTP class and the MELD score. All participants were followed for the development of ACLF and for all-cause mortality up to 28 days from enrolment.

We first checked continuous variables for normality with the Shapiro-Wilk test; results are reported as numbers and percentages for categorical variables, and since most continuous measures departed from a normal distribution, we used the median (interquartile range) alongside the mean±SD for reference. Group comparisons accordingly relied on the Mann-Whitney U test for continuous variables and on the chi-square test or Fisher's exact test for categorical variables, with Fisher's test used whenever an expected cell count fell below five. To identify the plasma cystatin C threshold that best predicted 28-day mortality, we constructed a receiver operating characteristic (ROC) curve and selected the cut-off that maximised the Youden index (sensitivity + specificity − 1); its area under the curve (AUC) was compared with that of the CTP and MELD scores, each computed in the same direction (death as the positive outcome), using DeLong's test. Significance was set at a two-sided p-value below 0.05, and all computations were carried out in SPSS Statistics version 21.0 (IBM Corp., Armonk, NY, USA).

## Results

A total of 100 patients with acutely decompensated liver cirrhosis, recruited over 18 months, were included in the analysis. The mean (SD) age was 48.8 (10.1) years (median 48.5, range 29-76), with the largest proportion of participants (34%) in the 41-50-year age group; 82 (82%) were men. Alcohol was the commonest aetiology of cirrhosis (75 patients, 75%), followed by hepatitis B (10, 10%), hepatitis E (5, 5%) and hepatitis C (3, 3%); none of the participants was seropositive for HIV. On presentation, ascites was documented in 93 patients (93%), haematemesis/malena in 90 (90%), icterus in 89 (89%), hepatic encephalopathy in 88 (88%), pedal oedema in 64 (64%) and pallor in 61 (61%). Most participants were classified as CTP class C (79%), followed by class B (17%) and class A (4%); the median CTP score was 11 (IQR 10-12). The median MELD score was 34.3 (IQR 32.2-37.1), and 64% of participants had a MELD score of 30-39. Acute-on-chronic liver failure developed in 41 patients (41%), and 44 patients (44%) died within 28 days of enrolment. The overall mean (SD) plasma cystatin C was 3.57 (3.72) mg/L (median 2.16, IQR 1.13-4.03); 77 patients (77%) had a level above 1.11 mg/L. The association between clinical features and 28-day mortality is shown in Table 1.

**Table 1:** Association between clinical features and 28-day mortality in patients with acutely decompensated liver cirrhosis (total n = 100) *A p-value <0.05 is considered statistically significant. χ²: Pearson chi-square statistic (df=1); ≈ denotes an approximate value. †: Fisher's exact test (used when an expected cell count was <5) does not yield a test statistic; only the p-value is reported.

Clinical feature	Mortality present n (%) (n=44)	Mortality absent n (%) (n=56)	Test statistic	p-value
Glasgow Coma Scale (GCS) ≤7	8 (18.2)	7 (12.5)	χ²≈0.62	0.430
Ascites	42 (95.5)	51 (91.1)	—†	0.461
Hepatic encephalopathy	39 (88.6)	49 (87.5)	χ²≈0.03	0.862
Haematemesis/malena	40 (90.9)	50 (89.3)	—†	1.000
Pallor	30 (68.2)	31 (55.4)	χ²≈1.70	0.192
Icterus	43 (97.7)	46 (82.1)	—†	0.021*
Pedal oedema	31 (70.5)	33 (58.9)	χ²≈1.42	0.233
Alcohol-related aetiology	30 (68.2)	45 (80.4)	χ²≈1.95	0.163
Hepatitis B positive	5 (11.4)	5 (8.9)	—†	0.745
Hepatitis C positive	2 (4.5)	1 (1.8)	—†	0.581
Hepatitis E positive	4 (9.1)	1 (1.8)	—†	0.166

Icterus was significantly more frequent among non-survivors (p=0.021). Other clinical and aetiological features did not differ significantly between those who died and those who survived. Laboratory parameters associated with 28-day mortality were compared using the Mann-Whitney U test, as none were normally distributed (Table 2).

**Table 2:** Comparison of laboratory parameters between patients with and without 28-day mortality (total n = 100) Only mean±SD, not median (IQR), was available in the source data for parameters other than cystatin C because the Mann-Whitney U test is rank-based and its validity does not depend on which summary statistic is displayed. All continuous variables were non-normally distributed (Shapiro-Wilk test) and were compared using the Mann-Whitney U statistic (reported for the mortality-present group; maximum possible value = 2464). The categorical cystatin C comparison used the chi-square (χ²) statistic (df=1). ≈: Denotes an approximate value; ‡: U>1706 reflects only that p<0.001; the exact value is more extreme; *A p-value<0.05 is considered statistically significant

Parameter	Mortality present, mean±SD (n=44)	Mortality absent, mean±SD (n=56)	Test statistic	p-value
Platelet count (×10⁹/L)	108.4 ± 60.1	99.4 ± 130.1	U≈1598	0.011*
Blood urea (mg/dL)	89.6 ± 74.1	53.4 ± 36.3	U≈1677	0.002*
Serum creatinine (mg/dL)	2.32 ± 2.12	1.13 ± 0.80	U>1706‡	<0.001*
Plasma cystatin C (mg/L)	5.49 ± 4.70	2.06 ± 1.54	U=1925	<0.001*
Cystatin C >1.11 mg/L, n (%)	40 (90.9)	37 (66.1)	χ²=8.58	0.003*
Total bilirubin (mg/dL)	8.79 ± 7.30	5.84 ± 4.99	U≈1586	0.014*
Direct bilirubin (mg/dL)	4.42 ± 4.69	3.21 ± 4.20	U≈1545	0.030*
Indirect bilirubin (mg/dL)	5.20 ± 4.51	3.22 ± 3.02	U≈1590	0.013*

Platelet count, blood urea, serum creatinine, plasma cystatin C, and total, direct, and indirect bilirubin were all significantly higher among non-survivors (p<0.05 for all). Plasma cystatin C level did not differ significantly between patients with and without ACLF, either as a continuous variable (mean (SD) 4.31 (4.31) vs 3.05 (3.19) mg/L; p=0.271) or when dichotomised at 1.11 mg/L (83% vs 73% of patients above this cut-off; p=0.240); the strength of association was small (point-biserial r=0.17). In contrast, plasma cystatin C was strongly and significantly higher among patients who died within 28 days than among survivors (mean (SD) 5.49 (4.70) vs 2.06 (1.54) mg/L; median 3.70 vs 1.35 mg/L; Mann-Whitney U=1925, p<0.001), with a large effect size (point-biserial r=0.46). Among the 44 patients who died, 40 (91%) had a cystatin C level above 1.11 mg/L, compared with 37 of 56 survivors (66%; χ²=8.58, p=0.003). On ROC curve analysis, plasma cystatin C predicted 28-day mortality with an area under the receiver operating characteristic curve (AUROC) of 0.781 (95% CI 0.688-0.874; p<0.001) (Figure 1).

**Figure 1:**
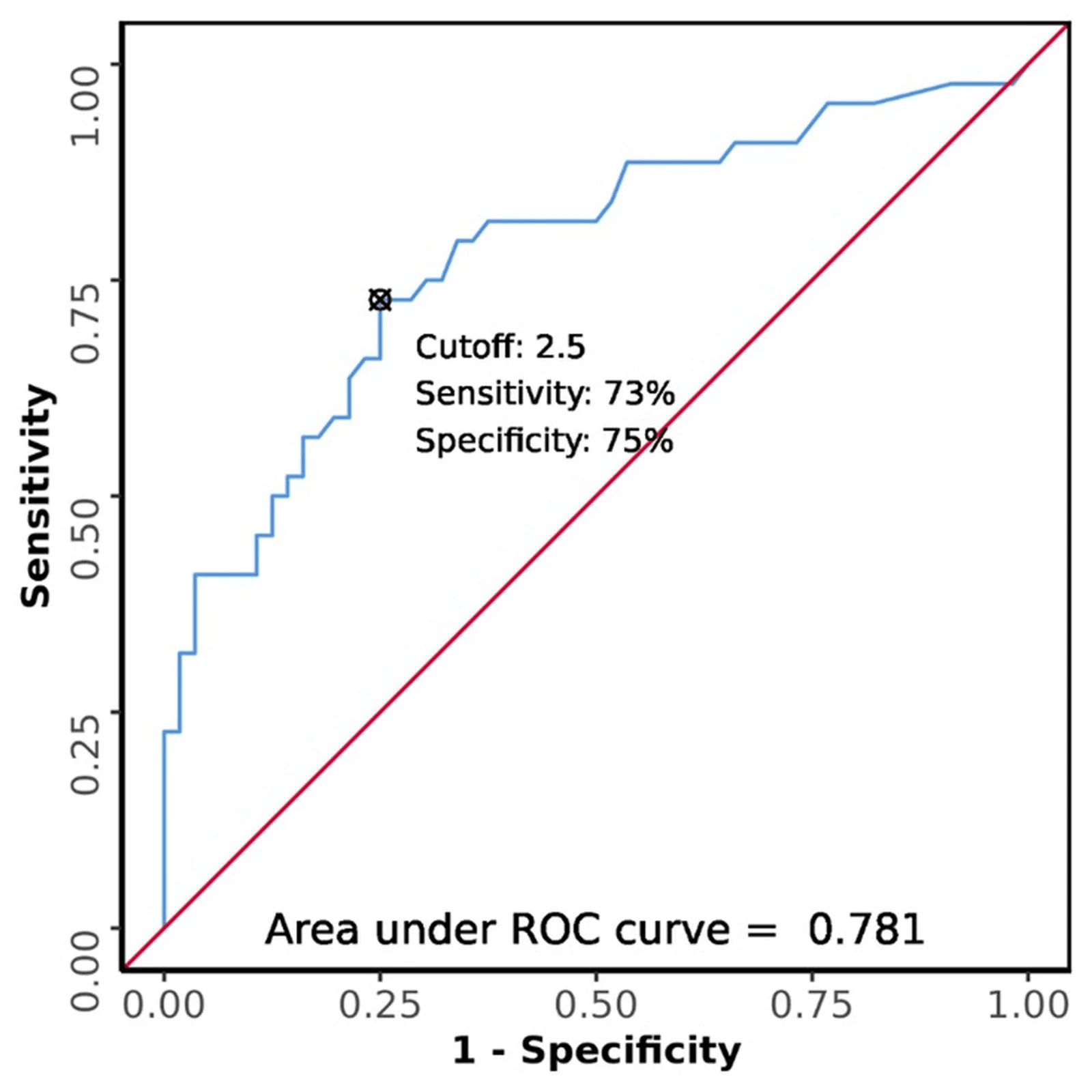
The ROC curve for plasma cystatin C in predicting 28-day mortality in patients with acutely decompensated liver cirrhosis The AUROC was 0.781 at a cut-off of ≥2.5 mg/L; sensitivity was 73%, and specificity was 75%. ROC: Receiver operating characteristic; AUROC: Area under the receiver operating characteristic curve

A cut-off of ≥2.5 mg/L gave a sensitivity of 73% (95% CI 57-85), specificity of 75% (95% CI 62-86), positive predictive value of 70% (95% CI 54-82), negative predictive value of 78% (95% CI 64-88) and diagnostic accuracy of 74% (95% CI 64-82); the odds ratio for 28-day mortality at this cut-off was 7.15 (95% CI 2.95-17.35). Diagnostic performance of cystatin C, CTP score, and MELD score for predicting 28-day mortality is compared in Table 3 and Figure 2. On DeLong's test, the AUROC for cystatin C was significantly higher than for the CTP score (0.506, 95% CI 0.392-0.620) or MELD score (0.508, 95% CI 0.392-0.624) (p<0.001 for both), while both the CTP and MELD scores did not differ significantly from each other (p=0.898).

**Table 3:** Diagnostic performance of plasma cystatin C, CTP score, and MELD score in predicting 28-day mortality (total n = 100) On DeLong's test, the AUROC for cystatin C was significantly higher than for the CTP score and MELD score (p<0.001 for both). The CTP and MELD scores did not differ significantly from each other (p=0.898). †A p-value for the null hypothesis that AUROC = 0.5; *A p-value <0.05, considered statistically significant; CTP: Child-Turcotte-Pugh; MELD: Model for End-Stage Liver Disease; AUROC: area under receiver operating characteristic curve

Predictor	AUROC (95% CI)	p-value†	Sensitivity (%)	Specificity (%)
Cystatin C (cut-off ≥2.5 mg/L)	0.781 (0.688-0.874)	<0.001*	73	75
CTP score	0.506 (0.392-0.620)	0.916	7	100
MELD score	0.508 (0.392-0.624)	0.892	23	88

**Figure 2:**
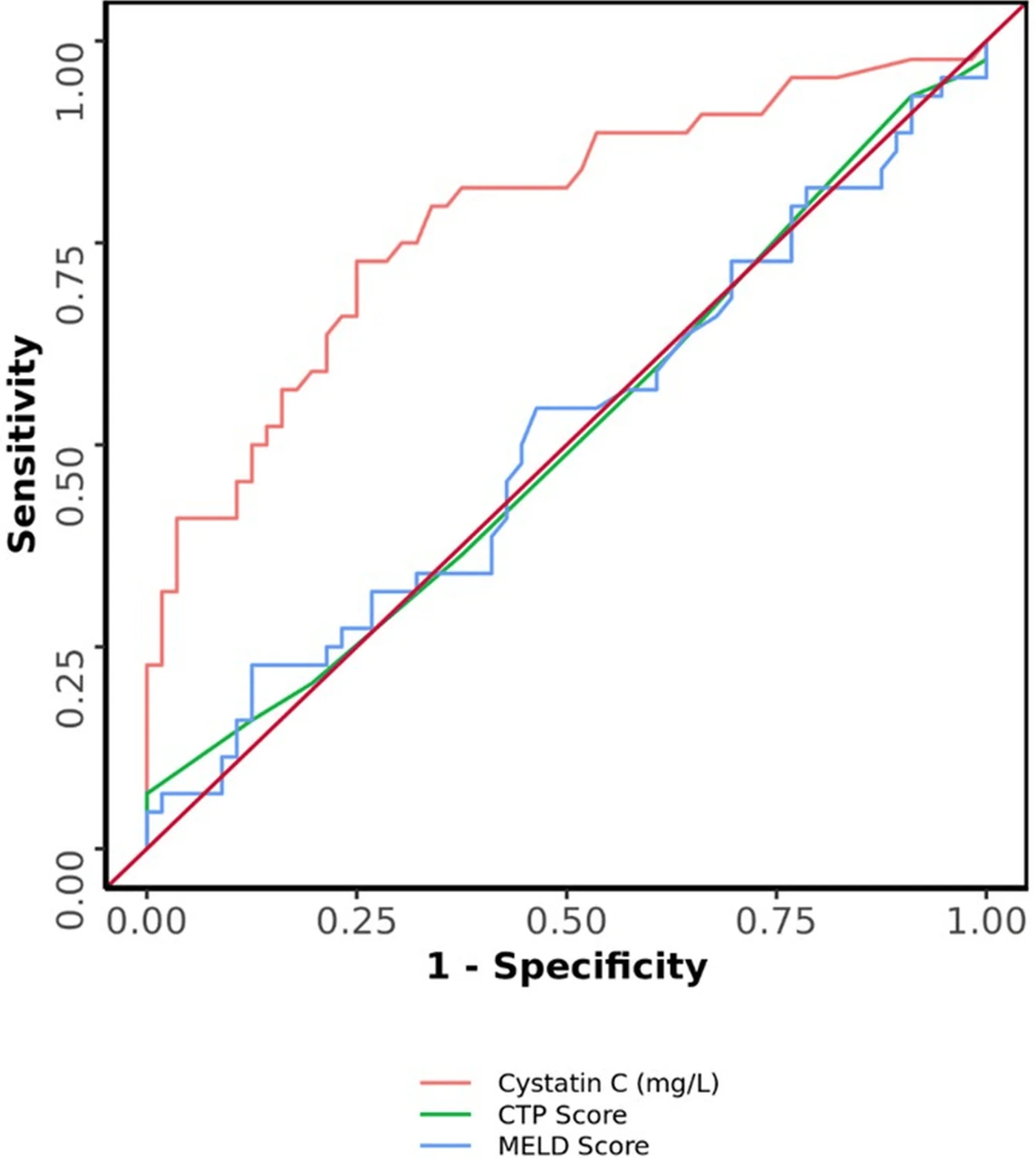
Comparative ROC curves for plasma cystatin C, CTP score, and MELD score in predicting 28-day mortality in patients with acutely decompensated liver cirrhosis ROC: Receiver operating characteristic; CTP: Child-Turcotte-Pugh; MELD: Model for End-Stage Liver Disease

## Discussion

In this prospective cohort of 100 patients with acutely decompensated liver cirrhosis, plasma cystatin C was significantly elevated in patients who died within 28 days of admission and discriminated 28-day mortality substantially better than the conventional CTP and MELD scores, with a proposed cut-off of 2.5 mg/L yielding 73% sensitivity and 75% specificity. The cystatin C level, however, did not differ significantly between patients with and without ACLF, suggesting that in this cohort it performed better as a marker of short-term mortality risk than as a discriminator of ACLF itself.

The prevalence of ACLF in our cohort (41%) is comparable to that reported in other prospective cohorts of acute decompensation of cirrhosis [7,11]. Our finding that plasma cystatin C rises significantly in patients who die within 28 days corroborates a large multicentre cohort in which baseline cystatin C independently predicted 90-day mortality, renal dysfunction, hepatorenal syndrome and ACLF [7], as well as reports from hepatitis B virus-related ACLF and decompensated cirrhosis in which a higher cystatin C level was associated with increased short-term mortality, and in which combining cystatin C with total bilirubin improved prediction of survival beyond conventional severity scores [11,12]. Cystatin C has similarly been reported to identify acute kidney injury earlier than creatinine and to independently predict outcome in other cohorts of cirrhosis, with proposed cut-offs in a range comparable to ours [13-15]. Unlike some of these reports, we did not find a significant association between cystatin C level and ACLF status itself; this may reflect differences in the criteria used to define ACLF, in disease severity, or in sample size across studies. The limited discrimination we observed for the CTP and MELD scores is in keeping with reports that conventional severity scores can perform inconsistently for short-term mortality prediction across ACLF cohorts and that other markers have sometimes outperformed them [16]. It may also reflect the referral bias of a tertiary-care centre, where nearly four-fifths of our participants were already in CTP class C, narrowing the range over which these scores could discriminate outcome.

A few limitations temper these findings. The work was carried out at one tertiary-care centre with no liver-transplant programme, and none of our participants were transplanted during follow-up. This probably contributed to the relatively high mortality we observed and limits how far the results can be extended to centres where transplantation is available. As an observational study, it can establish association but not causation, and our cohort was predominantly male and alcohol-related with a high proportion of CTP class C disease, which may limit generalisability to milder or aetiologically different populations. The sample size, while adequate for the pre-specified diagnostic-accuracy calculations, limited subgroup analysis by aetiology or organ-failure pattern; we also relied on univariable comparisons and ROC analysis rather than multivariable regression, so we cannot exclude confounding by variables such as age or bilirubin, and the proposed cystatin C cut-off requires external, ideally multivariable, validation before clinical use. Strengths include the prospective design, pre-specified sample-size calculation, and head-to-head comparison of cystatin C with two established prognostic scores in the same cohort using the same outcome definition; this is consistent with reports that cystatin C-based measures can add incremental value for the early detection of renal impairment even in milder disease [17].

## Conclusions

In this cohort, plasma cystatin C measured within 48 hours of admission was associated with a greater risk of death during the subsequent 28 days and showed better mortality discrimination than the CTP or MELD scores. It did not, however, distinguish ACLF status. Confirmation in larger multicentre cohorts is needed before the proposed 2.5 mg/L threshold can be applied clinically. If validated, cystatin C may provide a practical adjunct to established prognostic assessment in acutely decompensated cirrhosis.
